# Estrogenic Activity of Coffee Constituents

**DOI:** 10.3390/nu11061401

**Published:** 2019-06-21

**Authors:** Ryoiti Kiyama

**Affiliations:** Dept. of Life Science, Faculty of Life Science, Kyushu Sangyo Univ. 2-3-1 Matsukadai, Higashi-ku, Fukuoka 813-8503, Japan; kiyama.r@ip.kyusan-u.ac.jp; Tel.: +92-673-5240; Fax: +92-673-5092

**Keywords:** coffee constituents, phytoestrogens, estrogenicity, signal transduction pathway, estrogen assay

## Abstract

Here, the constituents of coffee with estrogenic activity are summarized by a comprehensive literature search, and their mechanisms of action for their physiological effects are discussed at the molecular and cellular levels. The estrogenic activity of coffee constituents, such as acids, caramelized products, carbohydrates, lignin, minerals, nitrogenous compounds, oil (lipids), and others, such as volatile compounds, was first evaluated by activity assays, such as animal tests, cell assay, ligand-binding assay, protein assay, reporter-gene assay, transcription assay, and yeast two-hybrid assay. Second, the health benefits associated with the estrogenic coffee constituents, such as bone protection, cancer treatment/prevention, cardioprotection, neuroprotection, and the improvement of menopausal syndromes, were summarized, including their potential therapeutic/clinical applications. Inconsistent results regarding mixed estrogenic/anti-estrogenic/non-estrogenic or biphasic activity, and unbeneficial effects associated with the constituents, such as endocrine disruption, increase the complexity of the effects of estrogenic coffee constituents. However, as the increase of the knowledge about estrogenic cell signaling, such as the types of specific signaling pathways, selective modulations of cell signaling, signal crosstalk, and intercellular/intracellular networks, pathway-based assessment will become a more realistic means in the future to more reliably evaluate the beneficial applications of estrogenic coffee constituents.

## 1. Introduction

Coffee is the third most abundant beverage in the world after water and tea, and is prepared by brewing roasted coffee beans; most often of the *Coffea arabica* (arabica coffee) and *C. canephora* (robusta coffee) species [1]. Its consumption has been increasing worldwide (162 million bags in the 2017 to 2018 period [2]) following the increase in its trade because of increasing needs based on taste, aroma, and health benefits. Coffee constituents, except water, are classified into the following materials: Acids, caramelized products, carbohydrates, lignin, minerals, nitrogenous compounds, oil (lipids), and others, such as volatile compounds (Table 1). Dried green coffee beans contain carbohydrates (59–62%), lipids (10–16%), proteins (10%), chlorogenic acids (7–10%), minerals (4%), aliphatic acids (2%), caffeine (1–2%), trigonelline (1%), and free amino acids (<1%), but roasting coffee beans reduces the amounts of carbohydrates, proteins, chlorogenic acids, and free amino acids [3] and increases those of alkaloids (mostly caffeine), minerals, oil, and aliphatic acids [4]. In contrast, there is no change in the amount of lignin [4]. As a result, roasting coffee beans changes their bioactivity, such as the induction of apoptosis [5] and alteration of gene expression [6].

Coffee contains hundreds of biologically active compounds, which result in different outcomes on human health, as revealed by epidemiological and clinical studies. For example, an inverse association between coffee consumption (3 to 4 cups/day) and all-cause mortality was observed, in addition to lower risks for cardiovascular diseases, such as coronary heart disease; congestive heart failure; hypertension and stroke; neurodegenerative diseases, such as Parkinson’s and Alzheimer’s diseases; liver diseases, such as hepatic steatosis and fibrosis; inflammatory diseases; and cancer [16,17,18,19,20,21]. Therapeutic outcomes of coffee were also observed, such as improvements in diabetes, metabolic syndrome, depression, obesity, and asthma control [20,22], and in slowing the progression of sarcopenia and promoting the regeneration of injured muscle [23]. Moreover, improvements can be observed in many tissues/organs, such as bone, heart, kidney, liver, lung, the nervous system, and the reproductive system/endometrium [24,25]. However, coffee may increase the risks of anxiety; insomnia; headaches; tremulousness; palpitations and hypertension, especially for heavy drinkers; the risk of fracture in women; and the risk of low birth weight and preterm birth when consumed during pregnancy due to the high caffeine content or the lack of appropriate metabolic enzymes [17,20,21,26,27]. On the other hand, studies in humans and animal models have produced controversial results about the safety and beneficial roles of caffeine, which may be due to the population, type and dose of caffeine, and low statistical power [28]. The accumulation of data from epidemiological and clinical studies and their meta-analysis gives more clear views with more statistical stability, such as the contribution of smoking to the association between coffee consumption and risk of hypertension [21], and the association of coffee consumption with all-cause/cardiovascular disease mortality [29], although there are limitations to such analyses, attributable to genetic variations among the individuals investigated, their habitual changes, and the duration effects of coffee consumption [29].

The beneficial effects of coffee could be mediated by varying mechanisms, such as inducing autophagy, improving insulin sensitivity, stimulating glucose uptake, slowing the progression of sarcopenia, and promoting the regeneration of injured muscle [23], and pathways, such as the AMP-activated protein kinase (AMPK) pathway for metabolic control [30] and the Nrf2/antioxidant regulatory element (ARE) pathway for the oxidative stress response [26]. Anti-angiogenic and anti-inflammatory properties of coffee could be partly mediated by the inhibition of cyclooxygenase-2 (COX-2) expression and monocyte chemoattractant protein-1 (MCP-1) secretion [31]. The effects of coffee are attributable to biologically active compounds, such as caffeine, diterpenes, chlorogenic acids, and melanoidins, although their amounts vary depending on the coffee species, roasting degree, brewing method, and serving size [32], and the nutritional constituents, such as milk and sugar, added to coffee may cause additional effects [22]. On the other hand, coffee contains potentially harmful compounds, such as acrylamide, which is formed during the process of roasting at high temperatures by the Maillard reaction and may have carcinogenic activity [33].

A number of natural chemicals have been reported to exhibit estrogenic activity, where effects, such as physiological/endocrinological, neurological, developmental, and behavioral effects, are combined unexpectedly or intentionally with hormone activity [34]. Estrogenic chemicals are classified by structure, such as phenolics, anilines, carboranes, indoles, metalloestrogens, perfluorinated compounds, phthalates, polycyclic aromatic hydrocarbons, and terpenes/terpenoids, or by the usages and effects, such as food additives/dietary supplements, pesticides, pharmacological estrogens, plasticizers, and pollutants [35]. An original idea that estrogen activity is initiated by binding estrogenic chemicals to the estrogen receptor (ER) via complex mechanisms involving several types of ERs and receptors other than ERs, along with signal crosstalk and intercellular/intracellular networks, compounds the complexity of estrogenic activity, resulting in more complex pathways and outcomes. Estrogen may also act as a mammary-gland carcinogen through estrogen metabolites, and the signaling pathways for cell proliferation and apoptosis [36], potentiating estrogenic chemicals to act similarly.

Assays to detect and evaluate estrogenic activity have been developed and classified according to their mechanisms: Animal tests, cell assays, ligand-binding assay, protein assays, reporter-gene assays, transcription assays, and the yeast two-hybrid assay (Table 2; for details, see Kiyama, 2017 [37]). Complex states of the estrogenic activity of chemicals partly originate from the methods or assays because they sometimes give inconsistent results, which is likely due to the inconsistencies between assays or conditions, such as temperature, concentrations, and time periods, and even between researchers employing the same protocols.

Estrogenic activity is mediated by many molecular mechanisms and cell signaling pathways, such as angiogenesis, ErbB/HER, mitogen-activated protein kinase (MAPK), nuclear receptor, and ubiquitin/proteasome signaling pathways, and resulting in cell functions, such as apoptosis, autophagy, cell cycle/DNA damage/cytoskeletal formation, cellular metabolism, chromatin/epigenesis, development/differentiation, immunology/inflammation response, neurological diseases, and translational control [35].

Coffee contains a number of estrogenic constituents, although they have not been focused on previously. Here, estrogenic coffee constituents are summarized in detail to explore future applications to consider new products as food supplements or new medical applications based on the beneficial applications of the constituents.

## 2. Estrogenic Activity of Coffee Constituents

### 2.1. Estrogenic Activity of Coffee Constituents

The estrogenic activity of coffee constituents has been known for more than 80 years [38]. Although epidemiological studies suggested the toxicity and complications associated with coffee, an understanding of the constituents responsible for such effects or health-promoting effects has become of interest to explore their beneficial applications. Here, the estrogenic activity of coffee constituents is summarized according to their chemical type.

#### 2.1.1. Acids

**Figure 1:**
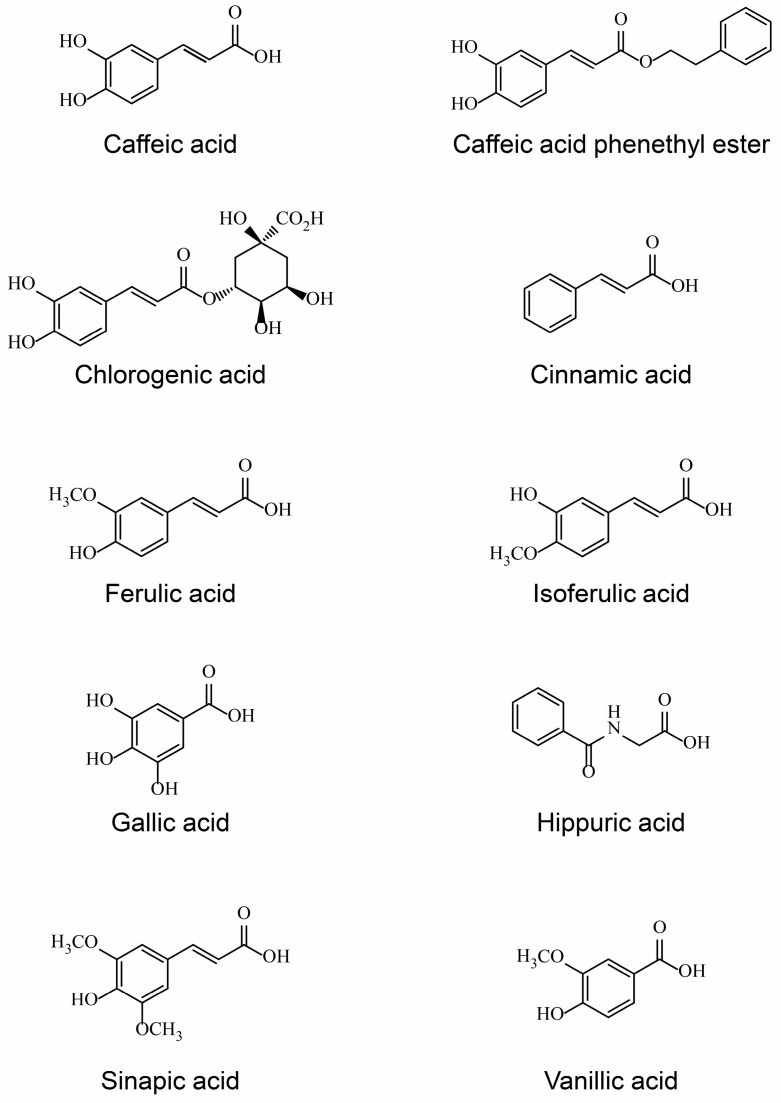

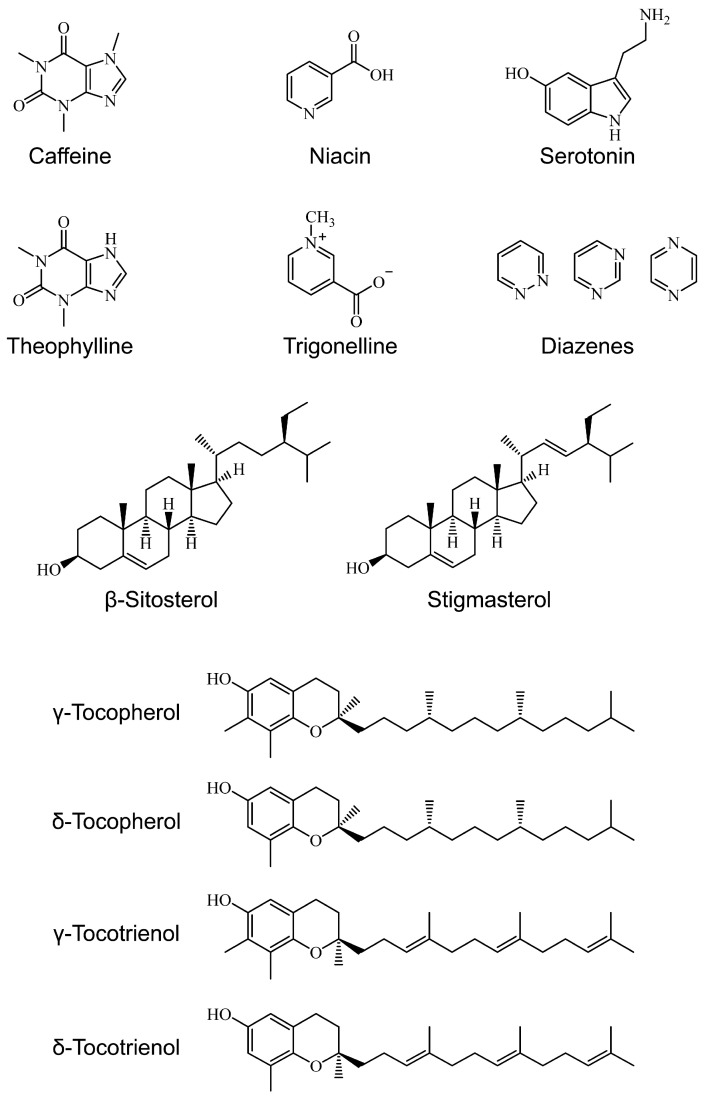
Structure of estrogenic coffee constituents.

Acids affect the taste and flavor of brewed coffee. Coffee contains many aromatic acids, such as chlorogenic acid and other quinic acid esters containing cinnamic acid/hydroxycinnamic acid, ferulic acid/isoferulic acid, gallic acid, hippuric acid, sinapic acid, and vanillic acid, and aliphatic acids, such as acetic acid, citric acid, lactic acid, and malic acid, some of which have been reported to exhibit estrogenic activity (Table 3, Figure 1).

Chlorogenic acid is an ester of caffeic acid and quinic acid, and was found to exhibit estrogenic activity [39], although weak [40] or too weak to be detected by other assays [41]. In contrast to quinic acid, which is a cyclic polyol unlikely to have estrogenic activity, caffeic acid is likely estrogenic, although this has not been confirmed [42,43]. Estrogen-dependent activities can sometimes appear as anti-estrogenic activity or modulator activities, as with selective estrogen receptor degraders (SERDs) and selective estrogen receptor modulators (SERMs), which are shown by a cinnamic acid ester/moiety [44,45] or caffeic acid phenethyl ester [46,47]. Furthermore, estrogenic/anti-estrogenic activity of caffeic acid is stronger when an aromatic ring is added by esterification with phenethyl alcohol [46,47]. Similarly, other acids found in coffee, such as ferulic acid/isoferulic acid [48], hippuric acid [49], sinapic acid [50], and vanillic acid [51,52], exhibited estrogenic/anti-estrogenic activity. Gallic acid is a phenolic acid with three hydroxyl groups and may therefore function in the estrogenicity, as observed for octyl gallate [53]; however, because of its simple structure, its estrogenic activity was unclear [54,55].

#### 2.1.2. Caramelized Products

Heating sucrose and other polysaccharides in the presence of amino acids, proteins, or acids produces caramels and colored products with a complex structure, which have been referred to as humic acids and melanoidins [11]. Although there were no reports regarding the estrogenic activity of humic acids from coffee, there were some regarding it from other sources, such as plant debris (Table 3). Humic substances are mainly composed of humic and fulvic acids, and lead to a decrease in estrogenic activity, as demonstrated in ER-dependent assays [56,57,58], although the mechanisms can be explained alternatively by direct interaction between them and estrogens through hydrogen bonding for chemicals highly rich in phenolic groups and/or π–π interactions for chemicals with greater aromaticity [59]. On the other hand, a synthetic humic substance containing dominant aromatic and quinoide structures exhibited estrogenicity [60]. Melanoidins are high molecular weight heterogeneous polymers that are formed through the Maillard reaction with sugars and amino acids, and add flavor to foods [61]. Many health-promoting effects, such as those against inflammation, diabetes, and hypertension, have been reported [3,32], although there have been no reports on the estrogenicity of melanoidins.

#### 2.1.3. Carbohydrates

Carbohydrates, such as sucrose, reducing sugars (arabinose, fructose, glucose, etc.), polysaccharides (araban, galactan, glucan, mannan, etc.), pectin (galacturonon), and glycosides (atractyligenin/atractyloside), are included in green and roasted coffee [11]. None of these, however, exhibited estrogenic activity, although sugar chains attached by glycosylation play a role in the strength of estrogenic activity. Glucose-conjugated isoflavones, for example, have weaker biological activity, including estrogenic activity, than the corresponding aglycone because they are highly polar and water-soluble, and are therefore hardly absorbed by the intestinal epithelium [95]. It is more difficult for such glucose-conjugated estrogenic chemicals to enter the cell to interact with nuclear ERs.

#### 2.1.4. Lignin

Lignin was identified as the insoluble residue of fiber in coffee and its amount does not change by roasting [15]. Lignin is the second most abundant plant polymer after cellulose, with complex structural characteristics, although it can be simplified into three basic building blocks, coumaryl alcohol, coniferyl alcohol, and sinapyl alcohol [96]. As these blocks all have a phenolic structure with potential hydrogen bonding and aromaticity, the ambivalent nature gives lignin potential estrogenic activity. Its degradation products thus may have estrogenic activity through ER-binding, whereas lignin was demonstrated to have anti-estrogenic activity through direct binding to estrogens, like humic acids (see above), by inhibiting the reabsorption of estrogens in the intestine, thereby decreasing plasma estrogen levels [67]. However, a cell-based estrogen assay revealed that methanol-soluble lignin, rich in the phenolic hydroxyl group, was not estrogenic [68]. Further studies are needed to understand the biological effects of lignin.

#### 2.1.5. Nitrogenous Compounds

Coffee contains several nitrogenous compounds, such as alkaloids (caffeine, theobromine, and theophylline), amino acid, nicotinic acid, nicotinamide, protein, trigonelline, and others (choline, serotonin amides, etc.), and some of them exert estrogenic activity (Table 3, Figure 1). Caffeine is a well-known constituent in coffee and is known for its health benefits due to its estrogenic effects; neuroprotective effects by estrogenic activity [69] and cancer chemopreventive effects by anti-estrogenic activity [70], for example. Other nitrogenous compounds, including their derivatives, such as nicotinic acid (niacin), serotonin amides, theophylline, and trigonelline, are also estrogenic/anti-estrogenic and may function in cardioprotection, neuroprotection, cancer chemoprevention, and bone protection [71,72,73,74,76,77]. Note that the effects vary depending on differences in signaling pathways and experimental conditions, and therefore result in estrogenic or anti-estrogenic effects (see Section 2.2).

#### 2.1.6. Oil

Many oils or lipids, such as diterpene alcohols/esters (cafestol and kahweol), phosphatides, squalene, sterols (campestanol, campesterol, cholesterol, citrostadienol, cycloeucalenol, 24-methylenelophenol, obtusifoliol, sitosterol, stigmastanol, and stigmasterol), tocopherols, triglycerides, tryptamine derivatives, and coffee wax (arachidic acid, behenic acid, 5-hydroxytryptamine, and lignoceric acid), are present in coffee [14]. Terpenes and terpenoids are an important category of components of coffee oil, and are associated with estrogenic activity (for a comprehensive review of estrogenic terpenes/terpenoids, see Kiyama, 2017 [97]). Sterols were reported to exhibit estrogenic/anti-estrogenic activity (Table 3, Figure 1), such as β-sitosterol [82,83,84,86] and stigmasterol [88]. Other types of terpenes included in coffee, such as γ/δ-tocopherol and γ/δ-tocotrienol, are also estrogenic/anti-estrogenic [89,90,91,92]. Lecithin is an important component of coffee and may exhibit numerous biological effects, although, due to its complex chemical composition, it has not been well-studied. Soy lecithin was found to be estrogenic and leads to the estrogenic activity of chocolate [81].

#### 2.1.7. Volatile Compounds and Mixtures of Chemicals

Volatile compounds included in coffee are cyclotene, ethylfuraneol. *N*-furfurylpyrrole, kahweofuran, isomaltol, maltol, oxazole, pyrazine, thiazole, thiophene, and diazenes (pyrazines, pyrimidines, and pyridazines) [15]. Due to their simple structures, no estrogenic activity has been detected, although their structures are known to affect estrogenic activity. The heterocyclic cores accommodated by diazenes and diazoles alter the geometry, integrity, and sizes of ER ligands, and ERα/ERβ selectivity [94].

Mixtures of chemicals include the extracts of coffee, which differ in solvents and conditions to make the extracts. The mixtures of chemicals after extraction of coffee with methanol or ethanol were reported to be estrogenic [38,93].

### 2.2. Estrogenic Cell-Signaling Pathways Associated with Coffee

Although the estrogenic cell-signaling pathways reported thus far for coffee constituents are those initiated by ERs (ERα and ERβ), other types of ERs, such as G-protein-coupled estrogen receptor 1 (GPER), estrogen-related receptors (ERRs), ER-α36, and ER-X, other receptors may regulate estrogen signals, as was reported for other estrogenic chemicals [35]. Once the receptor is activated by ligands, the signal is transduced via different signaling pathways in cells. Estrogenic signaling pathways include angiogenesis, ErbB/HER, MAPK, nuclear receptor and ubiquitin/proteasome signaling pathways, and the pathways related to apoptosis, including autophagy, cell cycle/DNA damage/cytoskeletal formation, cellular metabolism, chromatin/epigenesis, development/differentiation, immunology/inflammation response, neurological diseases, and translational control [98]. Coffee constituents were reported to activate estrogenic signaling pathways. For example, octyl gallate activated ERα/phosphoinositide 3-kinase (PI3K)/Akt signaling and ADAM10, an amyloid precursor protein processing enzyme, reduced amyloid-β production in a mouse model [53]. β-Sitosterol activates ERα/PI3K/GSK3β signaling to increase resistance to oxidative stress, which is beneficial for the treatment of neurodegenerative diseases, such as Alzheimer’s disease [86]. Chlorogenic acid is likely to induce osteoblast differentiation through the ERβ/Shp2/PI3K/Akt pathway [39]. Similarly, δ-tocotrienol may exert neuroprotective effects through the ERβ/PI3K/Akt pathway [92]. The involvement of MAPK pathways was reported for ferulic acid [64] and vanillic acid [51].

As estrogen signaling can be initiated by the binding of estrogenic chemicals with either ERα or ERβ, there are cases in which preference or selectivity was observed: 17β-estradiol (E_2_) and diazene motifs exhibit preferential binding to ERα [83,94], whereas caffeic acid phenethyl ester, chlorogenic acid and γ/δ-tocotrienol prefer ERβ (Table 3).

On the other hand, estrogen signaling can be initiated by the binding of chemicals with other types of ERs: Nicotinic acid with GPER [71], caffeic acid with membrane ERs [62], and vanillic acid with unspecified non-classical ERs [51]. Furthermore, other receptors have been reported to function in estrogen signaling by crosstalk, such as the insulin-like growth factor 1 receptor (IGF-1R) with the caffeic acid phenethyl ester [47] or caffeine [70], human epidermal growth factor receptor 2 (HER2) with ferulic acid [64], adenosine A_2A_ receptor with caffeine [69], peroxisome proliferator-activated receptor γ (PPARγ) with γ-tocopherol [89], and the serotonin (5-HT_1A_) receptors with serotonin [72] (Table 3). Upon the interaction between 5-HT_1A_ receptors and GPER on the plasma membrane, estrogen can induce desensitization of 5-HT_1A_ receptors for mood regulation [72].

Inconsistent or contradictory results for the estrogenicity of coffee constituents, such as hippuric acid, β-sitosterol, and trigonelline (Table 3), were observed among assays conducted by the same or different research groups [49,75,79,85]. This is attributable to the sensitivity of the assays [83], masking effects by other co-existing compounds [81], or the differences in doses/concentrations/purity of the chemicals [43,79,85], cell/tissue types used for the assays [76], or sources of ERs [49,80]. Note that some chemicals demonstrated biphasic effects, such as β-sitosterol, which is estrogenic at low doses and anti-estrogenic at high doses [82]. Although a standardized assay to evaluate estrogenicity may be an alternative [83], there may be intrinsic differences among the assays and molecules/cells/tissues/individuals used in the assays. Therefore, understanding the differences at the level of cell signaling pathways in addition to the principles of the assays, as discussed previously [35], is important.

## 3. Prospective of Estrogenic Coffee Constituents

### 3.1. Applications and Physiological Effects of Estrogens in Coffee

Although estrogen acts on the conventional endocrine target organs, such as the female reproductive tract, mammary glands, ovaries, and neuroendocrine system, estrogen also plays important roles in tissues, such as the bone, heart, and brain [99]. The physiological effects of caffeine, such as those on neuroprotection, cardioprotection, and digestive tract health, have been well documented [100], although attention has also been paid recently to the health benefits by other coffee constituents, especially those used to prevent diseases, such as neurodegenerative/cardiovascular diseases, metabolic syndromes, and cancer [17,18,32,101,102]. The physiological effects associated with estrogenic coffee constituents are closely related to the applications of the constituents and are discussed here.

#### 3.1.1. Bone Protection

Potential applications for bone protection and the treatment of osteoporosis were reported for caffeic acid, chlorogenic acid, and vanillic acid based on their estrogenic activity (Table 4, Figure 1). For example, caffeic acid and chlorogenic acid at high doses improved several symptoms in the skeletal system of ovariectomized rats, although the direct estrogen contribution was unclear or excluded [40,43]. In contrast, Zhou et al. demonstrated the improvement of estrogen deficiency-induced osteoporosis upon the administration of chlorogenic acid, as observed by bone mineral density and bone stem cell/osteoblast differentiation when specific cell-signaling pathways, such as Shp2/PI3K/Akt and cyclin D1 pathways, were examined [39]. They further speculated that the pathways involve the interaction of chlorogenic acid with ERβ [39]. Similar estrogenic effects on skeletal cells were observed for vanillic acid, although direct binding of vanillic acid with ERα or ERβ was not noted [51].

#### 3.1.2. Cancer Treatment and Prevention

Potential applications for cancer treatment and prevention were reported for cinnamic acid derivatives (caffeic acid phenethyl ester, caffeic acid/ferulic acid derivatives, cinnamic acid esters, and ferulic acid), terpenoids (β-sitosterol, γ/δ-tocopherol, and γ/δ-tocotrienol), and alkaloids (caffeine, theophylline, and trigonelline) (Table 4, Figure 1).

Several cinnamic acid derivatives are effective against cancer. For example, caffeic acid phenethyl ester increased the apoptotic effects of taxanes, i.e., docetaxel and paclitaxel, in prostate cancer cells by changing the expression of ERα and ERβ and modulating their signaling [47]. Lipophilic amides and esters of caffeic acid/ferulic acid exhibited cytotoxic effects on breast cancer cells by inducing apoptosis [63]. Furthermore, a mixture rich in cinnamic acid esters induced apoptosis of breast cancer MCF-7 cells by acting as an antagonist [44]. Although increasing lipophilicity may be important for some effects [63], ferulic acid alone can activate ERα signaling pathways and contribute to breast cancer treatment [64,66]. Note that the activation of ERα signaling by ferulic acid induces HER2 expression and alters cell metabolism, thereby increasing the sensitivity to anti-cancer agents, such as trastuzumab (Herceptin).

Terpenoids are an important category of food chemicals and include many estrogenic chemicals [97]. Sterols in plants, or phytosterols, belong to triterpenoids, which comprise six isoprene units, and serve as precursors of bioactive compounds and contribute to health-promoting effects as vitamins and anti-oxidants [103]. Although β-sitosterol, the most abundant phytosterol, stimulates tumor growth in vitro, dietary β-sitosterol reduced E_2_-stimulated tumor growth in mice, suggesting the consumption of β-sitosterol is beneficial for women with breast cancer [84]. Among eight different forms of vitamin E, i.e., four types of tocopherols and four types of tocotrienols, γ/δ-tocopherol and γ/δ-tocotrienol have been implied in cancer treatment and prevention. A mixture containing γ/δ-tocopherol showed decreased expression of ERα and suppression of E_2_-induced tumor growth, suggesting its use in breast cancer prevention [89,90]. Similar results for tumor cell growth inhibition were obtained for γ/δ-tocotrienol, where apoptosis was likely induced by γ/δ-tocotrienol through the ERβ signaling pathway [91].

Alkaloids, such as caffeine, theophylline, and trigonelline, were reported to be potential anti-cancer agents. Caffeine reduced the expression of ERα and IGF-1R, and inhibited both ERα-positive and -negative breast cancer cells through crosstalk between the receptors [70]. An 8-alkylthiothiated theophylline (TPBM) inhibited ERα binding to a consensus estrogen-responsive element (ERE), which resulted in the inhibition of ERα-mediated transcription and estrogen-dependent growth of tumor cells [74]. Trigonelline is a metabolic product of niacin and can be widely found in plants, including coffee. Trigonelline activated ER and stimulated the growth of estrogen-dependent breast cancer cells in vitro. However, there was no clear estrogenic activity in vivo or trigonelline did not compete against E_2_ in vitro, suggesting the activation to be mediated by a separate mechanism involving new signal mediators [62,77].

#### 3.1.3. Cardioprotection

Nicotinic acid (niacin) is the water-soluble vitamin B_3_ known for its beneficial effects for cardioprotection [104]. Niacin binds GPER and activates the GPER-mediated signaling pathways, including those potentially related to cardioprotection [71].

#### 3.1.4. Neuroprotection

Estrogenic activities of octyl gallate, β-sitosterol, and δ-tocotrienol have been considered to be useful to treat neurodegenerative diseases, such as Alzheimer’s and Parkinson’s diseases (Table 4). For example, octyl gallate, an important moiety for ER-binding in epigallocatechin-3-*O*-gallate, may activate amyloid-β processing through activation of ERα/PI3K/Akt signaling, and thereby reduce the amount of amyloid-β protein in mouse Alzheimer’s disease models [53]. β-Sitosterol prevented oxidative stress and lipid peroxidation in neuronal cells via ERα/PI3K/GSK3β signaling, suggesting the chemical is effective against Alzheimer’s disease [86]. In a mouse model of Parkinson’s disease, δ-tocotrienol was effective against the loss of dopaminergic neurons [92]. Caffeine and serotonin are also effective against Parkinson’s disease and depressive disorders, respectively, although the mechanisms may involve crosstalk between ERs and other receptors (dopamine and serotonin receptors) [69,72].

#### 3.1.5. Treatment of Menopausal Syndromes

Chemicals with estrogenic activity are useful agents to treat menopausal syndromes, such as those used in estrogen replacement therapy, and some coffee constituents, such as caffeic acid, caffeic acid phenethyl ester, ferulic acid/isoferulic acid, sinapic acid, stigmasterol, and theophylline, are potential candidates (Table 4, Figure 1). For example, caffeic acid phenethyl ester exhibited an affinity for ERβ and increased ERE-dependent transcription, although no estrogenic activity was observed in cell or uterotrophic assays, suggesting the chemical to be a potential SERM [46]. Among phenolic acids and phenolic esters found in the plant black cohosh, which has been used for the treatment of menopausal syndromes, ferulic and isoferulic acids were found to be slightly estrogenic [48]. Due to strong estrogenic activity without mutagenic activity, stigmasterol was considered to be a good candidate for estrogen replacement therapy [88]. Although theophylline did not significantly increase the uterine wet weight in a rat uterotrophic assay, it exhibited estrogen-binding, and increased the uterine RNA/protein amounts and uterine edema induced by E_2_ [73]. After menopause, estrogen deficiency may cause the development of obesity and metabolic disorders. Potential applications for the treatment of obesity and metabolic disorders caused by estrogen deficiency were reported for caffeic acid and sinapic acid, although it is unclear whether their estrogenic activity led to the observed outcomes [42,50].

#### 3.1.6. Endocrine Disruption

Excessive estrogen can potentially cause endocrine disruption, reproductive dysfunction, and other unfavorable effects. Such effects were reported for coffee extracts, hippuric acid, humic acid, lecithin, and β-sitosterol (Table 4). For example, coffee extracts have weak estrogenic activity, which may lead to adverse physiological effects in pregnant women [38,93]. Hippuric acid is a metabolite of phthalates, which are suspected to be endocrine disruptors, and exhibited weak estrogenic activity, although its contribution was marginal [49]. Aromatic humic substances are estrogenic and may cause endocrine disruption [60], whereas humic acids may exert anti-estrogenic effects, partly due to their direct binding to estrogen [56,57,58]. Among the constituents in foodstuffs, less characterized lecithin was found to have strong estrogenic activity, and it may cause toxic effects in adults and infants [81]. To understand the effects of environmental chemicals, a variety of chemicals were examined for estrogenic activity by several different assays, and β-sitosterol was found to be estrogenic [83].

### 3.2. Potentials of Future Applications

Pathway-based assessment of estrogenic activity has been focused on in several fields, such as environmental, toxicological, and pharmacological, where pathway-based assessment of estrogenic activity should not be considered as an alternative of outcome-based assessment, such as animal tests, but as a paradigm shift to a new mechanism-based assessment, providing sufficient predictability and variability [35]. Variations detected by pathway analysis are not limited to the level of cellular signaling pathways but include those at the levels of receptors, signal crosstalk, and intercellular networks (see Section 2.2). Moreover, additional variations may aid in applications as follows.

Variations in the estrogenic effects were found by altering the effects of the receptor functions, such as SERMs and SERDs, and some coffee constituents were reported to be associated with these modulators. SERMs are the substances that have differing agonist/antagonist activity among different tissues (such as between uterus and breast) via the regulation of receptor functions (e.g., coregulators’ selectivity) and/or selection of receptor subtypes (e.g., ERα/ERβ). Therefore, they differentially inhibit or stimulate estrogen activity in these tissues [105,106]. Several coffee constituents were reported to act as SERMs, such as caffeic acid phenethyl ester [46,47], cinnamic acid (moiety) [45], and diazenes (motifs) [94]. SERDs, on the other hand, are a class of substances that inhibit ER functions by binding to and degrading the ER [107]. Cinnamic acid (moiety) was also reported among SERDs [45,108].

Each estrogenic coffee constituent may exert additional activities that affect the variability. For example, coffee constituents act as inhibitors or activators of functions other than estrogen activity; caffeic acid is a selective inhibitor of 5-lipoxygenase [109], caffeic acid phenethyl ester is an inhibitor of NF-κB [110], cinnamic acid ester is an inhibitor of 17β-hydroxysteroid dehydrogenase [111], gallic acid is an activator of Tousled-like kinase 1 [112], γ-tocopherol is an inhibitor of PPAR-γ [113], and an unidentified chemical in coffee is an opiate receptor antagonist [114]. Some of these constituents can act on two or more pathways via crosstalk (see Section 2.2).

When benefits are expected, it is important to consider the potential risks. Although the health-promoting effects and therapeutic potential of coffee constituents have been described, unfavorable effects have been reported for some constituents. For example, caffeic acid at low doses and trigonelline exerted unfavorable effects on bone, such as estrogen-dependent decreases in bone mineralization and mechanical properties of bone [43,76]. Trigonelline, a natural component in green coffee beans and other unidentified compounds, was found to be mutagenic, especially after roasting [115]. Due to the estrogenic activity, there was concern as to whether trigonelline can stimulate the growth of estrogen-dependent cancer in vivo [75].

## 4. Conclusions

As the consumption of coffee is steadily increasing worldwide, there has been more interest in its health effects. Epidemiological and clinical studies revealed that moderate levels of coffee consumption do not result in detrimental outcomes (except some cases, such as pregnant women) but rather exert beneficial effects toward human health. As the interest in coffee is increasing, more products originating from coffee enriched with particular constituents have been created, which has prompted researchers to assess the effects of each constituent. Here, the constituents of coffee were classified into acids, caramelized products, carbohydrates, lignin, minerals, nitrogenous compounds, oil (lipids), and others (such as volatile compounds), and the chemicals in each classification were examined for estrogenic activity. Estrogen is one of the important hormones whose mechanisms of action have been extensively studied. Furthermore, there are a number of chemicals present in nature or industrial products/biproducts that mimic estrogen. From a comprehensive literature search, chemicals groups, such as phenolic acids, humic acids, lignin, alkaloids, terpenoids, and volatile compounds, have been reported in association with their estrogenic activity in addition to its physiological effects and/or mechanisms of action, which resulted in either beneficial effects/applications, such as bone protection, cancer treatment/prevention, cardioprotection, neuroprotection, and the improvement of menopausal syndromes, or unbeneficial effects, such as endocrine disruption. In addition to the increasing interest in the beneficial effects of coffee, pathway-based assessment of the effects, including estrogenic activity, will be more important in the future to more reliably evaluate epidemiological and clinical data and make beneficial applications.

## Figures and Tables

**Table 1:** List of major coffee constituents.

Chemical	Amount (%w/w)		
	Green ^a^	Roasted ^a^	Roasted ^b^
**Acids**			
Chlorogenic acids	5.5–8.0	1.2–2.3	2.5
Quinic acid			0.7
Aliphatic acids (citric acid, malic acid, lactic acid, acetic acid, etc.)	1.5–2.0	1.0–1.5	1.5
**Caramelized products**	0	16.0–17.0	23.2
Humic acids, melanoidins, etc.			
**Carbohydrates**			
Sucrose			0
Reducing sugars (glucose, fructose, arabinose, etc.)			0.3
Polysaccharides (glucan, galactan, araban, mannan, etc.)	50.0–55.0	24.0–39.0	32.0
Pectin (galacturonon)			3.0
**Lignin**			2.0
**Minerals**	3.0–4.2	3.5–4.5	5.0
K, Ca^2+^, Mg^2+^, etc.			
**Nitrogenous compounds**			
Alkaloids (caffeine, theobromine, theophylline, etc.)	0.9–1.2	~1.0	1.4
Amino acid	2.0	0	0
Nicotinic acid			0.015
Protein	11.0–13.0	13.0–15.0	10.0
Trigonelline	1.0–1.2	0.5–1.2	0.4
Others (choline, serotonin amides, etc.)			
**Oil**	12.0–18.0	14.5–20.0	18.0
Diterpene alcohols/esters, phosphatides, sterols, tocopherols, triglycerides,			
Tryptamine derivatives, etc.			
**Others**			
Volatile compounds (pyridines, quinolines, pyrazines, pyrroles, etc.)			0.1

The list of chemicals was made from the information in Clarke (1987) [4] and Viani (1988) [7], where typical compositions of green and roasted arabica coffee are shown (^a^ Smith, 1985 [8]; ^b^ Clarke, 1987 [4]). The chemicals included in the respective category are detailed in Clifford (1985) [9] (chlorogenic acid), Woodman (1985) [10] (aliphatic acids), Trugo (1985) [11] (carbohydrates), Clarke (1985) [12] (minerals), Macrae (1985) [13] (nitrogenous compounds), Folstar (1985) [14] (oil), and Dart and Nursten (1985) [15] (volatile compounds).

**Table 2:** Assays used to detect estrogenic activity.

Assay Category (Symbol)/Description	Example ^a^
**Animal test (A)**	
Animal tests quantitate reproductive, developmental and behavioral effects in animals.	Medaka assay
	Zebrafish assay
	Rainbow trout assay
	Rodent uterotrophic assay
	*Xenopus* assay
**Cell assay (C)**	
Cell assays quantitate cell growth and proliferation.	Cell counter assay
	Cell density/viability assay (SRB assay/AlamarBlue assay/MTS assay/MTT assay/WST-8 assay)
	Dye exclusion method (Trypan blue assay)
	E-screen assay
	Flow cytometry
**Ligand-binding assay (L)**	
Ligand-binding assays quantitate the receptor–ligand interaction.	Assay with ERs in cells
	Assay with ERs in tissues (uterus, etc.)
	Assay with recombinant ERs
	Assay with Venus fluorescent protein
	Competitive enzyme immunoassay
	Fluorescence polarization assay
	Molecular docking
	QSAR
**Protein assay (P)**	
Protein assays quantitate protein amounts and functions.	ChIP assay
	ELISA
	Immunoassay (ICC, IHC)
	Western blotting (ERα/ERβ)
	Western blotting (Akt/ERK)
**Reporter-gene assay (R)**	
Reporter-gene assays quantitate the transcription upon ligand-dependent binding of the receptor to an estrogen response element on DNA.	CALUX assay
	GFP-based assay
	Luciferase-based assay
	MVLN cell assay
	YES assay
**Transcription assay (T)**	
Transcription assays quantitate the transcription of ER or marker genes.	DNA microarray assay
	Northern blotting
	RT-PCR
**Yeast two-hybrid assay (Y)**	
Yeast two-hybrid assays quantitate the ligand-dependent interaction between the receptor and the transcriptional activator.	GAL4-based assay
	Whole hERα-based assay

^a^ The examples are adapted from Kiyama (2017) [37]. For details of estrogenic chemicals analyzed by each assay, see Kiyama and Wada-Kiyama (2015) [35]. Abbreviations: CALUX: chemically activated luciferase expression; ChIP: chromatin immunoprecipitation; ELISA: enzyme-linked immunosorbent assay; ER: estrogen receptor; ERK: extracellular signal-regulated kinase; GFP: green fluorescent protein; ICC: immunocytochemistry; IHC: immunohistochemistry; MTS: (3-(4,5-dimethylthiazol-2-yl)-5-(3-carboxymethoxyphenyl)-2-(4-sulfophenyl)-2H-tetrazolium); MTT: 3-(4,5-dimethylthiazol-2-yl)-2,5-diphenyltetrazolium bromide; QSAR: quantitative structure-activity relationship; RT-PCR: reverse transcription polymerase chain reaction; SRB: sulforhodamine B; WST-8: (2-(2-methoxy-4-nitrophenyl)-3-(4-nitrophenyl)-5-(2,4-disulfophenyl)-2H-tetrazolium); YES: yeast estrogen screen.

**Table 3:** Estrogenic coffee constituents.

Chemical	Receptor/Pathway	Estro-Genicity ^a^	Function or Subject	Reference (Assay ^b^)
**Acids**				
Caffeic acid	ER	E	Menopausal syndrome/Obesity	Zych et al., 2009 [42] (A)
Caffeic acid	ER	E/A	Bone protection	Folwarczna et al., 2015 [43] (A)
Caffeic acid	ER	N	Breast cancer/Chemoprevention	Nunes et al., 2017 [62] (C, T)
Caffeic acid phenethyl ester	ERβ	S	Menopausal syndrome	Jung et al., 2010 [46] (A, L, R)
Caffeic acid phenethyl ester	ERα/β (crosstalk)	S	Prostate cancer/Chemoprevention	Tolba et al., 2013 [47] (C, P, T)
Caffeic acid/Ferulic acid derivatives	ER	A	Breast cancer/Chemoprevention	Serafim et al., 2011 [63] (A, C, P)
Chlorogenic acid	ER	N	Menopausal syndrome	Innocenti et al., 2007 [41] (R)
Chlorogenic acid	ER	E/N	Bone protection	Folwarczna et al., 2009 [40] (A)
Chlorogenic acid	ERβ	E	Osteoporosis/Bone protection	Zhou et al., 2016 [39] (A, C, P)
Cinnamic acid esters	ER	A	Breast cancer/Chemoprevention	Hostanska et al., 2004 [44] (C)
Cinnamic acid (moiety)	ERα	S	Breast cancer/Chemoprevention	Kieser et al., 2010 [45] (L, P, T)
Ferulic acid	ERα (crosstalk)	E	Breast cancer/Chemoprevention	Chang et al., 2006 [64] (C, P, T)
Ferulic acid	ER	N	Menopausal syndrome	Wen et al., 2011 [65] (R, T)
Ferulic acid	ERα	E	Breast cancer/Chemoprevention	Belkaid et al., 2016 [66] (C, P)
Ferulic acid/Isoferulic acid	ER	E	Menopausal syndrome	Stromeier et al., 2005 [48] (C)
Gallate (Octyl)	ERα	E	Alzheimer’s disease	Zhang et al., 2013 [53] (A, P)
Gallic acid	ER	N	Endocrine disruption	Miller et al., 2001 [54] (R)
Gallic acid	ERα/β	N	Phytoestrogen/Health benefits	Mallavadhani et al., 2006 [55] (L)
Hippuric acid	ER	E	Endocrine disruption	Picard et al., 2001 [49] (C, L)
Sinapic acid	ER	E	Metabolic disorders	Zych et al., 2018 [50] (A, P)
Vanillic acid	ERα/β	E	Osteoporosis/Bone protection	Xiao et al., 2014 [51] (C, L, P, T)
Vanillic acid	ERα	A	Benign prostatic hyperplasia	Jung et al., 2017 [52] (A, C, P, T)
**Caramelized products**				
Humic acids	ER	A	Endocrine disruption	Janosek et al., 2007 [56] (R)
Humic acids	ER	A	Endocrine disruption	Tang et al., 2014 [58] (Y)
Humic acids	ER	A	Endocrine disruption	Bedard et al., 2014 [57] (P, R)
Humic substance (synthetic)	ER	E	Endocrine disruption	Lutz et al., 2005 [60] (A, T)
**Carbohydrates**				
None				
**Lignin**				
Lignin	ER	A	Enterohepatic circulation	Arts et al., 1991 [67] (L)
Lignin (methanol)	ER	N	Endocrine disruption	Nakamura et al., 2001 [68] (C)
**Nitrogenous compounds**				
Caffeine	ER (crosstalk)	A	Parkinson’s disease	Xu et al., 2006 [69] (A)
Caffeine	ERα (crosstalk)	A	Breast cancer/Chemoprevention	Rosendahl et al., 2015 [70] (C, P)
Nicotinic acid (Niacin)	GPER	E	Cardioprotection	Santolla et al., 2014 [71] (C, L, P, T)
Serotonin	GPER (crosstalk)	E	Depressive disorder	Li et al., 2013 [72] (A, P)
Theophylline	ER	E	Estrogenic response	Steinsapir et al., 1982 [73] (A, L)
Theophylline derivative (TPBM)	ERα	A	Breast cancer/Chemoprevention	Mao et al., 2008 [74] (C, P, R)
Trigonelline	ER	E	Carcinogenesis/Phytoestrogen	Allred et al., 2009 [75] (C, L, R, T)
Trigonelline	ER	E/A	Bone protection	Folwarczna et al., 2014 [76] (A)
Trigonelline	ER	E	Colon cancer/Chemoprevention	Yoo and Allred, 2016 [77] (C, R, T)
**Oil**				
Campesterol/β-Sito-sterol/Stigmasterol	ER	N	Endocrine disruption	Baker et al., 1999 [78] (A, L, R)
Campesterol/β-Sito-sterol/Stigmasterol	ERα	N	Endocrine disruption	Procházková et al., 2017 [79] (R)
Citrostadienol	ER	N	Reproductive dysfunction	Mellanen et al., 1996 [80] (A, C, T)
Lecithin (soy)	ERα	E	Endocrine disruption	Behr et al., 2011 [81] (R)
β-Sitosterol	ER	B	Feminization	Rosenblum et al., 1993 [82] (A, L)
β-Sitosterol	ERα/β	E	Endocrine disruption	Gutendorf and Westendorf, 2001 [83] (C, L, R)
β-Sitosterol	ER	E	Breast cancer/Chemoprevention	Ju et al., 2004 [84] (A, C, T)
β-Sitosterol	ER	N	Cell proliferation	Shappell et al., 2012 [85] (C)
β-Sitosterol	ERα	E	Alzheimer’s disease	Shi et al., 2013 [86] (C, P)
Stigmastanol	ER	N	Endocrine disruption	Monteverdi and Di Giulio, 1999 [87] (C, P)
Stigmasterol	ER	E	Estrogen replacement therapy	Boldrin et al., 2013 [88] (R)
γ-Tocopherol (mixture)	ERα	A	Cancer/Chemoprevention	Smolarek et al., 2013 [89] (A, P, T)
γ/δ-Tocopherol	ER	A	Cancer/Chemoprevention	Bak et al., 2017 [90] (A, P, T)
γ/δ-Tocotrienol	ERβ	E	Cancer/Chemoprevention	Comitato et al., 2009 [91] (L, P, T)
δ-Tocotrienol	ERβ	E	Parkinson’s disease	Nakaso et al., 2016 [92] (A, P)
**Other (volatile components and mixtures of chemicals)**				
Coffee extract (ethanol)	ER	E	Endocrine disruption	Kitts, 1987 [38] (A, L)
Coffee extract (80% methanol)	ER	E	Endocrine disruption	Takamura-Enya et al., 2003 [93] (R)
Diazenes	ERα/β	S	Breast cancer/Chemoprevention	Ghosh et al., 2003 [94] (L, R)

Chemicals include derivatives of the chemicals listed and those found in green coffee. The categories listed are shown in Table 1. ^a^ Activity: anti-estrogenic (A), biphasic (B), estrogenic (E), not estrogenic (N), or SERM (S). ^b^ Abbreviations for the assays used to detect estrogenic activity are: animal test (A), cell assay (C), ligand-binding assay (L), protein assay (P), reporter-gene assay (R), transcription assay (T), and yeast two-hybrid assay (Y) (see Table 2). ER: estrogen receptor; GPER: G protein-coupled estrogen receptor 1; SERM: selective estrogen receptor modulator.

**Table 4:** Applications and physiological effects of estrogenic coffee constituents.

**Bone protection/Osteoporosis (Estrogenic)**
Caffeic acid, chlorogenic acid, vanillic acid
**Cancer treatment and prevention (Estrogenic/anti-estrogenic)**
Caffeic acid phenethyl ester, caffeic acid/ferulic acid derivatives, caffeine, cinnamic acid esters, diazenes, ferulic acid, β-sitosterol, theophylline, γ/δ-tocopherol, γ-tocopherol (mixture), γ/δ-tocotrienol, trigonelline
**Cardioprotection (Estrogenic)**
Nicotinic acid (niacin)
**Endocrine disruption/Reproductive dysfunction (Mostly estrogenic)**
Coffee extract, hippuric acid, humic acids, lecithin, β-sitosterol
**Menopausal syndrome/Endocrine disease (Estrogenic)**
Caffeic acid, caffeic acid phenethyl ester, ferulic acid/isoferulic acid, sinapic acid, stigmasterol, theophylline
**Neuroprotection (Mostly estrogenic)**
Caffeine, gallate (octyl), serotonin, β-sitosterol, δ-tocotrienol

Note that chemicals are listed in a representative category (see Table 3).
